# Arousal and exposure duration affect forward step initiation

**DOI:** 10.3389/fpsyg.2015.01667

**Published:** 2015-11-03

**Authors:** Daniëlle Bouman, John F. Stins, Peter J. Beek

**Affiliations:** Department of Human Movement Sciences, Faculty of Behavioural and Movement Sciences, Vrije Universiteit Amsterdam, MOVE Research Institute Amsterdam, Netherlands

**Keywords:** forward gait initiation, affect, emotion, arousal, exposure duration

## Abstract

Emotion influences parameters of goal-directed whole-body movements in several ways. For instance, previous research has shown that approaching (moving toward) pleasant stimuli is easier compared to approaching unpleasant stimuli. However, some studies found that when emotional pictures are viewed for a longer time, approaching unpleasant stimuli may in fact be facilitated. The effect of viewing duration may have modulated whole-body approach movement in previous research but this has not been investigated to date. In the current study, participants initiated a step forward after viewing neutral, high-arousal pleasant and high-arousal unpleasant stimuli. The viewing duration of the stimuli was set to seven different durations, varying from 100 to 4000 ms. Valence and arousal scores were collected for all stimuli. The results indicate that both viewing duration and the arousal of the stimuli influence kinematic parameters in forward gait initiation. Specifically, longer viewing duration, compared to shorter viewing duration, (a) diminished the step length and peak velocity in both neutral and emotional stimuli, (b) increased reaction time in neutral stimuli and, (c) decreased reaction time in pleasant and unpleasant stimuli. Strikingly, no differences were found between high-arousal pleasant and high-arousal unpleasant stimuli. In other words, the valence of the stimuli did not influence kinematic parameters of forward step initiation. Instead the arousal level (neutral: low; pleasant and unpleasant: high) explained the variance found in the results. The kinematics of forward gait initiation seemed to be reflected in the subjective arousal scores, but not the valence scores. So it seems arousal affects forward gait initiation parameters more strongly than valence. In addition, longer viewing duration seemed to cause diminished alertness, affecting GI parameters. These results shed new light on the prevailing theoretical interpretations regarding approach motivation in the literature, which warrants further examination in future research.

## Introduction

Emotion and action are strongly intertwined, but exactly how they are coupled, is not yet fully understood. Emotion theorists (Frijda, 1986; Bradley et al., 2001; Lang and Bradley, 2010; Phaf et al., 2014) argue that emotions activate or prime motivational tendencies (both defensive and appetitive), like approach and avoidance behavior.

Evidence of the emotion-action link has been found in behavioral experiments that have attempted to operationalize approach/avoidance behaviors in a variety of ways. In general, the results indicated that it is easier to organize an approach movement in response to a pleasant item, and easier to organize an avoidance movement in response to an unpleasant item, compared to alternate pairings. According to some authors, this effect constitutes evidence for the “motivational direction hypothesis” (MDH; Bradley et al., 2001) and has been consistently found in manual reaction time tasks (e.g., Chen and Bargh, 1999; Eder and Rothermund, 2008).

In most pertinent experiments, visual stimuli have been used to induce emotional states. These stimuli are typically selected from the International Affective Picture System (IAPS; Lang et al., 2005) and involve pictures varying in valence (pleasantness) and arousal, based on the assumption that emotions can be classified along these two dimensions (Lang et al., 2005). Other emotional stimuli can involve facial expressions (Stins et al., 2011) or sounds (Komeilipoor et al., 2013).

In the last decade or so, novel ways to study approach/avoidance behaviors have been adopted. Traditional responses involved pushing (avoidance) and pulling (approach) a lever (Chen and Bargh, 1999), but other responses may include discrete manual (forward/backward) responses such as keypresses (e.g., De Houwer et al., 2001), moving a doll forward or backward (e.g., Lavender and Hommel, 2007), deflecting a joystick (e.g., Eder and Rothermund, 2008), and whole-body movement paradigms (e.g., Naugle et al., 2011).

The whole-body movement paradigm has been motivated by the desire to incorporate more ecologically valid behavioral measures, that may resemble more closely actual bodily motion toward or away from an emotional cue (e.g., Koch et al., 2009), compared to one-degree-of-freedom manual responses.

Within the whole-body movement paradigm, three different but related methods are used: quiet standing (e.g., Horslen and Carpenter, 2011), (2) gait initiation involving a single step (GI; e.g., Stins and Beek, 2011), and (3) locomotion (e.g., Naugle et al., 2011). In all these paradigms evidence has been found for the proposition that postural control can be affected by emotion. In the current experiment we adopted the second method, focusing on the control of forward gait initiation. The reason is that this paradigm allows us to study most clearly directional effects of emotion, i.e., the ease with which a forward (approach) movement is organized and executed.

GI is the phase between quiet standing and walking and involves the neural control of balance and timing of muscle activation. It is generally divided in two distinct processes: a postural phase and an execution phase (Brenière et al., 1987). In forward (single step) GI, the center of pressure (COP; application point of the ground reaction forces) is initially decoupled from the center of mass (COM) and moves behind the COM, causing a forward acceleration (Brenière et al., 1987). GI consists of the actor lifting the swing leg (so that the body weight is transferred to the stance leg) and swinging it forward, using the stance leg for push off. The swing leg lands some distance anterior, and the stance leg is pulled forward and lands next to the other leg. Note that these events result in a characteristic pattern of ground reaction forces that can thus be identified in the COP trajectory.

It has been widely reported that emotional states are reflected in the COP trace, and can influence gait initiation parameters like velocity, step length, and reaction time (e.g., Gélat et al., 2011; Naugle et al., 2011; Stins and Beek, 2011; Stins et al., 2015).

Experiments within the whole-body movement paradigm with emotional stimuli have shown many interesting effects of affective cues on goal-directed movement, including effects that seem consistent with the MDH, i.e., faster whole-body movement initiation in the direction of pleasant stimuli (e.g., Stins and Beek, 2011; Yiou et al., 2014; Stins et al., 2015). The opposite effect (faster whole-body movement initiation *away* from unpleasant stimuli) has not been found to date (Stins and Beek, 2011; Stins et al., 2014; Yiou et al., 2014).

Some studies found an unexpected effect that seemed to contradict the basic tenet of the MDH, namely that it was sometimes easier to execute a forward step toward an *unpleasant* picture. Naugle et al. (2011) found, in some conditions, empirical evidence for such an effect using the GI paradigm. Stins et al. (2015) reasoned that some of the effects reported by Naugle et al. (2011) might have been due to fact that forward GI was in response to stimulus *disappearance*, i.e., at stimulus offset. In other words, participants had to withhold their step for the duration the picture was presented on the screen. Most other studies to date, in contrast, asked participants to produce a response at stimulus *onset*. To this end, Stins et al. (2015) directly compared two paradigms; GI at the offset of the cue (disappearance) and GI at the onset of the cue (i.e., stepping forward as soon as the cue appeared on the screen). Only with the onset condition the expected effect was found, namely faster forward GI with pleasant compared to unpleasant stimuli. At stimulus offset the opposite effect was found, similar to Naugle et al. (2011), which again seemed to contradict the MDH.

At present, the reason for the offset GI effect, contrasting the MDH, is unclear, but it could be the case that the effect is modulated by the viewing duration of the stimuli. Namely, one of the differences between the two conditions in the experiment of Stins et al. (2015) is the amount of time the participants were looking at the picture, before having to initiate their step. In the onset condition, viewing time before initiating the step coincided with the response time. However, in the offset condition the viewing time (i.e., duration the picture was shown on the screen) varied randomly between 3 and 5 s, prior to GI.

Viewing duration has not been directly manipulated in GI paradigms before. However, based on the studies mentioned previously and brain imaging studies on the temporal dynamics of emotional processing, viewing duration of emotional stimuli warrants further investigation within the whole-body movement paradigm. When initiating a step at onset (e.g., Stins and Beek, 2011) the viewing duration before step initiation is relatively short, namely as long as the reaction time. In these onset paradigms, pleasant and unpleasant stimuli affect GI differentially, generally in support of the MDH. This differentiation between emotional stimuli has been mirrored in studies on the temporal dynamics of emotional processing. For example, both Esslen et al. (2004) and Smith et al. (2003) found differences in early temporal activation in the brain in response to various emotional categories.

In both Naugle et al. (2011) and Stins et al. (2015), the offset conditions caused a relatively longer viewing duration of the stimuli (3–5 s in Stins et al., 2015 and 2–4 s in Naugle et al., 2011). Both studies showed differential effects of emotional stimuli in GI as well, but apparently contradicting the MDH. This differentiation at a later time in emotional processing is comparable to unique neural signatures found later in the stages of processing related to different emotional stimuli. For example, a comprehensive study of Dan-Glauser and Gross (2011) found that viewing of IAPS pictures (pleasant, unpleasant, neutral) for 8 s induced a complex temporal response pattern, involving cognitive, subjective, physiological, and facial expressive changes. Furthermore, Sabatinelli et al. (2009) found that a late amygdala response could be observed discriminating between neutral and high-arousal stimuli.

The timing of affective processing in the brain may modulate the coupling between emotion and action. In the current experiment we sought to systematically investigate the hypothesis that viewing duration influences the mechanics of forward step initiation. Instead of directly contrasting onset and offset conditions, we used only the offset condition whereby the viewing duration was controlled (i.e., independent of individual patterns of response time like in onset manipulations). Our hypothesis was that for short durations, participants would respond faster to pleasant pictures compared to unpleasant pictures (consistent with the MDH) but that this pattern would switch with longer durations, with participants responding faster to unpleasant pictures compared to pleasant pictures. We additionally tested the effect of emotion and duration on other key GI parameters related to step execution.

## Materials and Methods

### Participants

Thirty-two healthy individuals (18 females; Mean age = 23.4, SD = 3.0) participated in the experiment. The participants were screened for injuries of lower extremities and other injuries that prevented them from walking or standing properly. The experiment was approved by the local ethics committee and informed consent was signed by all participants prior to the experiment.

### Materials and Methods

Posturographic data were recorded using a custom-made strain gauge force plate (1 × 1 m; sampling frequency: 100 Hz). The force plate recorded forces with eight sensors; four measuring forces in the *z* direction, and two sensors each for the *x* and *y* directions. The data from these sensors were converted to forces in three directions (*Fx*, *Fy*, and *Fz*) from which moments (*Mx*, *My*, *Mz*) were calculated. The COP was then calculated using *Mx* and *My*. The COP represents the point of application of the ground reaction force (for details see Brenière et al., 1987).

The images were shown on a 55-inch monitor positioned 1.5 m in front of the participant at eye-level. Image offset was detected by a photodiode attached to the monitor (not visible to the participant), which was synchronized with the force plate recording. The stimuli were chosen from the IAPS (Lang et al., 2005). Only high-arousal pictures were used since previous research has revealed that only high-arousal pictures have discernible impact on gait initiation (Stins et al., 2015). Five picture categories were chosen from the IAPS: (1) erotica and (2) extreme sports (both pleasant, high-arousal), (3) mutilation and (4) threat (both unpleasant, high-arousal), and (5) neutral. From each picture category we selected 16 unique images^1^.

These picture are comparable to the high-arousal pictures used in previous research on emotion and GI (e.g., mutilation, attack/threat and erotica; Yiou et al., 2014; Stins et al., 2015). To ensure that the pictures were truly highly arousing, we made sure that the high-arousal pictures all had an arousal rating greater than 5 (i.e., above the median value on the SAM scale) according to the normative ratings reported by Lang et al. (2005). The average scores for the neutral, pleasant and unpleasant categories (both normative and experimental) are shown in Table 1. Additionally, the four high-arousal picture categories chosen have been classified as high-arousal pleasant and unpleasant categories by Bradley and Lang (2007; e.g., Figure 2.4, p. 36). Therefore, we feel confident that the stimulus set had the desired property.

**TABLE 1:** **Mean (+SD) for SAM scores of valence and arousal of both the normative scores from the IAPS manual and the scores from the current experiment**.

****	**Normative scores**	**Experiment**
**Valence**		
Neutral	4.86 (0.28)	5.00 (0.15)
Pleasant	6.80 (0.29)	6.48 (0.26)
Unpleasant	2.22 (0.30)	2.40 (0.36)
**Arousal**		
Neutral	2.75 (0.50)	1.46 (0.34)
Pleasant	5.96 (0.37)	3.98 (0.43)
Unpleasant	6.64 (0.36)	5.29 (0.52)

Participants filled out the 9-point Self-Assessment Manikin (SAM; Bradley and Lang, 1994) in order to rate each picture on the dimensions of valence and arousal. Higher scores on these scales indicate higher valence (i.e., pleasantness) and higher arousal. In addition, participants filled out the State-Trait Anxiety Inventory (STAI; Spielberger et al., 1983) to ensure that the group did not score high or low on anxiety, which may influence the results (Naugle et al., 2011).

### Procedure

**FIGURE 1:**
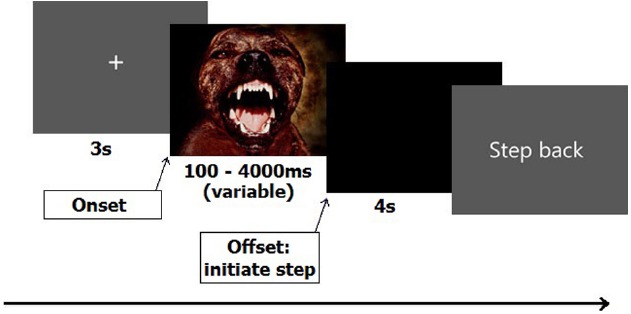
**Sequence of stimulus events for one trial**.

After signing the informed consent, participants filled out the STAI. Next, they stepped onto the force plate. Starting position was one of the corners, which was marked by a piece of white tape attached to the plate, in order to ensure that all participants started with their heels positioned in the same starting position. From this position participants had to initiate a step forward toward the opposite corner, which was closest to the monitor (cf. Stins et al., 2015). A 5-min practice session preceded the experiment. Each trial started with a 5 s on-screen message, instructing the participant to keep their feet at shoulder-width and look at the fixation cross. The fixation cross, which appeared directly after the instruction, stayed on screen for 3 s, after which one of the IAPS pictures appeared. The duration of the picture randomly varied among seven different durations ranging from 100 to 4000 ms (100, 300, 500, 1000, 2000, 3000, and 4000 ms). Participants were instructed to stand still and look at the picture until it disappeared from the screen, and then initiate a step forward as soon as possible. No instructions were given on step size or speed. All steps had to be initiated with the right leg and participants had to wait in their new position (closer to the screen) for 4 s until the instruction “step back” appeared on the screen. Participants then had 5 s to resume their original position and await the new trial. The sequence of stimulus events is shown schematically in Figure 1.

Pictures of each of the five different emotion categories were presented in separate blocks, each lasting about 5 min. The reason for separating the categories into blocks, rather than showing pictures from all categories randomly, is that valence of a given picture can influence processing of the immediately following affective stimulus (Gélat et al., 2011). These authors found that the COP of a given forward step was affected differently when the previous trial was pleasant, compared to when it was unpleasant. We therefore decided to present all pictures within the same emotion category in blocked fashion.

Within each block of trials, the duration of each picture was varied randomly. Each duration combined with a unique picture was presented twice within a block, resulting in 14 steps for each category block, and thus 70 steps in total. Two catch trials were added to each block, in which a large white cross was presented immediately after picture offset, indicating that participants did not have to step at all. These trials were included to keep participants alert. Between each block, participants were given the opportunity to rest and/or stretch their arms and legs, before continuing with the next block. The neutral block was shown first to all participants, and then the four subsequent emotional blocks were presented pseudo-randomly, ensuring that each block was presented an equal number of times in each order across participants. The number of trials per condition are shown in Table 2.

**TABLE 2:** **Number of trials per participant for each Duration × Emotion category condition**.

****	**Short**	**Medium**	**Long**
Neutral	6	4	4
Pleasant	12	8	8
Unpleasant	12	8	8

Short: 100, 300, and 500 ms. Medium: 1000 and 2000 ms. Long: 3000 and 4000 ms. Pleasant: erotica and sports picture categories. Unpleasant: mutilation and threat picture categories.

After the experiment, participants completed the SAM scale for all 80 pictures shown during the experiment. The pictures for the SAM scale were shown in a random order on a monitor and participants used paper and pencil for the ratings.

### Data Reduction

The COP time series and the raw force traces were rotated by 45° (due to the rotation of the force plate; see Stins et al., 2015), generating a new time-series with an anterior-posterior (AP) component in the direction of the screen, and a medio-lateral (ML) component for sideway excursions of the COP. The data were filtered using a 5-point moving average. The following GI parameters were analyzed (similar to Stins et al., 2015).

#### Reaction Time

The reaction time was determined as the time interval between picture offset (cue for GI) and the moment at which the force in the posterior direction exceeded 5 N.

#### APA Amplitude

The anticipatory postural adjustment (APA) was quantified as the distance in AP-direction between the initial position of the COP and the most posterior and lateral displacement of the COP in the direction of the right leg (sometimes labeled “S1”; see Naugle et al., 2011). The APA is related to the generation of forward momentum of the body to generate the desired step velocity by the end of the first step (Lepers and Brenière, 1995). Sixteen APA values were discarded due to an atypical initial displacement in the anterior direction instead of the posterior direction.

#### Step Size

The difference along the AP-axis between the initial position of the COP and the final position after completing the step was determined as the step size.

#### Peak Velocity

The peak velocity was quantified as the value of the maximum velocity of the COP trace during forward step. This generally coincides with the mid-swing phase of the swing leg (i.e., the right leg). Velocity was determined by numeric differentiation of the COP trace in the AP-ML plane.

**FIGURE 2:**
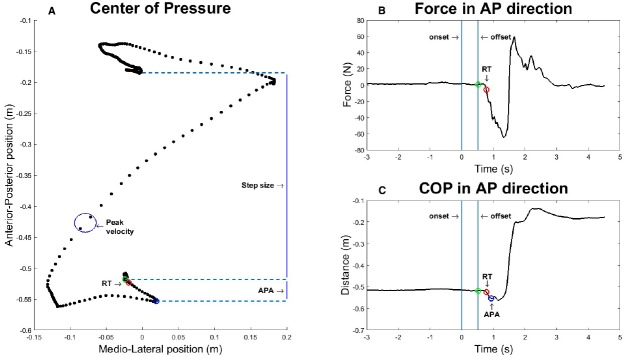
**Graphic representation of how the four dependent variables are extracted from a representative step after a stimulus duration of 500 ms. (A)** Center of Pressure (COP) in anterior-posterior (AP) and medio-lateral (ML) direction over time. **(B)** Force exerted by the participant in AP direction. **(C)** COP in AP direction over time.

Calculation of these four values is shown schematically in Figure 2, which displays a representative step.

### Statistical Analysis

The pleasant categories (extreme sports and erotica) and unpleasant categories (threat and mutilation) were grouped together, creating three different emotion conditions: neutral, pleasant and unpleasant. Furthermore, the seven different durations were averaged and grouped as short (100, 300, and 500 ms), medium (1000 and 2000 ms) and long (3000 and 4000 ms) durations. The short and long groups are in accordance with the study by Stins et al. (2015), which examined stimulus duration indirectly (onset/offset: 3000–5000 ms).

All data analysis was done using IBM SPSS Statistics version 21. The four dependent GI variables were analyzed using a 3 (duration: short, middle, and long) × 3 (emotion: neutral, pleasant, and unpleasant) multivariate repeated measures analysis of variance (MANOVA) to control for type-I error (see Naugle et al., 2011; Stins et al., 2015). If significant, follow-up analyses of the four dependent variables were performed using separate 3 × 3 repeated measures analyses of variance (ANOVAs). Greenhouse-Geisser correction was used if the assumption of sphericity was violated. Significant effects were examined using *post hoc* paired samples *t*-tests with Bonferroni correction. Separate ANOVAs (emotion: neutral, pleasant, unpleasant) were performed on both the arousal and the valence SAM scores. Alpha was set to 0.05.

With respect to effect sizes, we report the partial eta squared (${\text{η}}_{\text{p}}^{\text{2}}$) for the MANOVA results. Additionally, for the ANOVAs we report not only the common ${\text{η}}_{\text{p}}^{\text{2}}$, but also the generalized eta squared (${\text{η}}_{\text{G}}^{\text{2}}$). This latter measure is not yet widely adopted, but authors such as Lakens (2013) claim that it is a more robust measure than partial eta squared. For details on calculation and theory, see Bakeman (2005), Olejnik and Algina (2003), and Lakens (2013).

Effect sizes are also reported for the *post hoc* paired-samples *t*-tests. The recommended effect size for the *post hoc* paired-samples *t*-tests is Hedges’ g_average_ (g_av_; see Lakens, 2013 for details on theory and calculation). A common language (CL) effect size, introduced by McGraw and Wong (1992), is also presented to provide a more intuitive metric of effect size (Lakens, 2013). CL can be interpreted as the probability (%) that a person scores higher on one mean compared to the other, after controlling for individual differences.

## Results

We removed 121 trials (out of 2240; 5.4%) from the analysis for the following reasons: (a) stepping with the left leg, (b) excessive COP movement during picture presentation. This was based on visual inspection of the histogram of the SD of the movement in AP direction. The cutoff was set at 10 mm, which resulted in the removal of 1.4% of all trials, (c) stepping too early (RT < 150 ms), and (d) stepping too late (RT > 1000 ms).

### Questionnaires

The mean scores for all questionnaire measures are reported in Table 3. The scores for both the STAI trait and state anxiety scores were comparable to those reported by Naugle et al. (2011), indicating that our sample was similar in that regard. We did not separate the scores for males and females, as previous research has found that gender does not influence kinematic parameters (Naugle et al., 2011).

**TABLE 3:** **Mean (+ SD) of participant characteristics**.

Age	23.3 (3.0)
STAI-trait	34.9 (6.2)
STAI-state	30.2 (5.7)

### SAM; Valence

There was a main effect of emotion category, *F*(1.29,39.87) = 415.02, *p* < 0.001, ${\text{η}}_{\text{p}}^{\text{2}}$ = 0.93, ${\text{η}}_{\text{G}}^{\text{2}}$ = 0.91. *Post hoc* analysis showed that valence was significantly different for all three categories, with unpleasant pictures being scored lower (less pleasant) than both neutral [*t*(31) = 21.59, *p* < 0.001, Hedges g_av_ = 5.36, CL effect size = 99%] and pleasant pictures [*t*(31) = 21.57, *p* < 0.001, Hedges g_av_ = 6.33, CL effect size = 99%]. Pleasant pictures were scored as significantly more pleasant than neutral pictures [*t*(31) = –13.84, *p* < 0.001, Hedges g_av_ = 2.97, CL effect size = 99%]. These valence scores show a similar pattern to the ones reported by Yiou et al. (2014).

### SAM; Arousal

There was a main effect of emotion category, *F*(2,62) = 153.73, *p* < 0.001, ${\text{η}}_{\text{p}}^{\text{2}}$ = 0.83, ${\text{η}}_{\text{G}}^{\text{2}}$ = 0.64. Follow-up analysis showed that the three emotion categories differed significantly with respect to arousal, with neutral stimuli being significantly lower compared to both pleasant stimuli [*t*(31) = –10.91, *p* < 0.001, Hedges’ g_av_ = 2.19, CL effect size = 97%] and unpleasant stimuli [*t*(31) = –18.14, *p* < 0.001, Hedges’ g_av_ = 3,48, CL effect size = 99%]. Ratings of pleasant stimuli were significantly lower than ratings of unpleasant stimuli as well [*t*(31) = –5.851, *p* < 0.001, Hedges’ g_av_ = 0,90, CL effect size = 85%]. These arousal scores are similar to those reported by Yiou et al. (2014), with unpleasant pictures showing a higher arousal compared to pleasant pictures. For an overview of all SAM scores, see Table 1.

### Gait initiation Parameters

**FIGURE 3:**
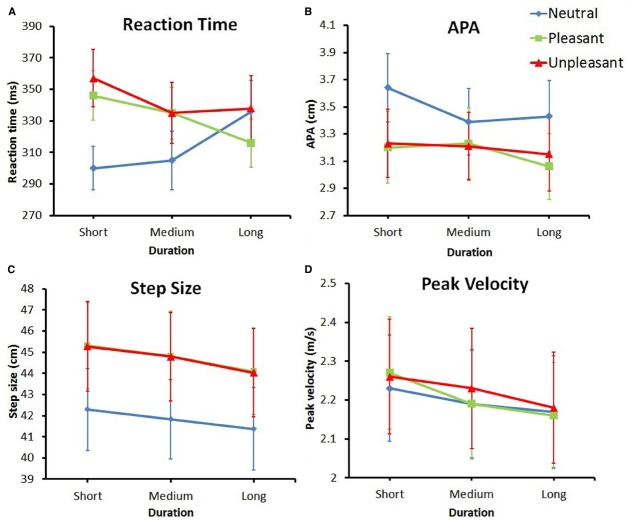
**Plotted means (+ Standard Errors) for all dependent variables. (A)** Reaction time. **(B)** Anticipatory Postural Adjustment. **(C)** Step size. **(D)** Peak velocity.

The MANOVA revealed a significant effect of emotion, *F*(8,24) = 5.69, *p* < 0.001, ${\text{η}}_{\text{p}}^{\text{2}}$ = 0.66, a significant effect of duration, *F*(8,24) = 3.08, *p* < 0.05, ${\text{η}}_{\text{p}}^{\text{2}}$ = 0.51, and a significant interaction of duration and emotion, *F*(16,16) = 3.02, *p* < 0.05, ${\text{η}}_{\text{p}}^{\text{2}}$ = 0.75. Means and standard deviations for all variables and conditions are reported in Table 4 and the means (+ Standard Errors) are plotted in Figure 3.

**TABLE 4:** **Mean (+ SD) of the GI variables for all three duration and emotion categories**.

****	****	**Short**	**Medium**	**Long**
RT (ms)	Neu	300 (77)	305 (105)	336 (110)
	P	346 (89)	335 (93)	316 (87)
	U	357 (103)	335 (109)	338 (117)
APA (cm)	Neu	3.64 (1.42)	3.39 (1.38)	3.43 (1.48)
	P	3.20 (1.48)	3.23 (1.46)	3.06 (1.36)
	U	3.23 (1.43)	3.21 (1.41)	3.15 (1.53)
Step size (cm)	Neu	42.29 (10.97)	41.83 (10.68)	41.38 (11.03)
	P	45.31 (11.58)	44.81 (11.97)	44.10 (11.51)
	U	45.27 (12.00)	44.79 (11.84)	44.03 (11.87)
Peak velocity (m/s)	Neu	2.23 (0.77)	2.19 (0.78)	2.17 (0.82)
	P	2.27 (0.82)	2.19 (0.80)	2.16 (0.77)
	U	2.26 (0.84)	2.23 (0.88)	2.18 (0.81)

Neu, neutral; P, pleasant; U, unpleasant.

### Reaction Time

Reaction time is plotted in Figure 3A. The effect of emotion on RT, *F*(2,62) = 8.144, *p* < 0.01, ${\text{η}}_{\text{p}}^{\text{2}}$ = 0.21, ${\text{η}}_{\text{G}}^{\text{2}}$ = 0.015, indicated a difference in reaction time for the various emotion categories. This effect was superseded by the interaction between duration and emotion, *F*(4,124) = 9.25, *p* < 0.001, ${\text{η}}_{\text{p}}^{\text{2}}$ = 0.23, ${\text{η}}_{\text{G}}^{\text{2}}$ = 0.015. *Post hoc t*-tests showed multiple effects. First, the RT in response to neutral stimuli became significantly longer over time; RT in response to short duration neutral pictures was significantly smaller compared to long duration neutral pictures [*t*(31) = –2.86, *p* < 0. 01, Hedges g_av_ = 0.36, CL effect size = 69%].

In contrast to neutral stimuli, in response to both pleasant and unpleasant stimuli, the RT decreased over time. In unpleasant stimuli the RT decreased when comparing short to medium viewing duration [*t*(31) = 3.51, *p* < 0.01, Hedges g_av_ = 0.20, CL effect size = 73%] and in pleasant stimuli the RT decreased when comparing short to long viewing duration [*t*(31) = 4.23, *p* < 0.001, Hedges g_av_ = 0.32, CL effect size = 77%].

Furthermore, when viewing a picture for a short duration, participants responded significantly faster to neutral pictures compared to both pleasant [*t*(31) = –4.80, *p* < 0.001, Hedges g_av_ = 0.53, CL effect size = 80%] and unpleasant [*t*(31) = –5.05, *p* < 0.001, Hedges g_av_ = 0.60, CL effect size = 81%] stimuli. When viewing a picture for a medium duration, participants only responded faster to neutral pictures compared to unpleasant pictures [*t*(31) = –2.61, *p* < 0.05, Hedges g_av_ = 0.27, CL effect size = 68%]. After viewing a picture for a long time, there was no difference in RT for the different emotion categories.

### Anticipatory Postural Adjustment

The APA values are plotted in Figure 3B. The ANOVA showed a main effect for emotion, *F*(1.27,39.31) = 5.67, *p* < 0.05, ${\text{η}}_{\text{p}}^{\text{2}}$ = 0.77, ${\text{η}}_{\text{G}}^{\text{2}}$ = 0.15, indicating a difference in APA amplitude between different emotion categories, regardless of duration. The comparison between neutral and pleasant stimuli and the comparison between neutral and unpleasant stimuli (with the Bonferroni-corrected alpha set at 0.05/3 = 0.01667) were both marginally significant [*t*(31) = 2.54, *p* = 0.017, and *t*(31) = 2.41, *p* = 0.022, respectively]. Looking at the data, a general trend appeared to exist in that neutral stimuli resulted in larger APAs compared to both pleasant and unpleasant stimuli.

### Step Size

Step size is plotted in Figure 3C. The main effect of emotion, *F*(1.49,46.14) = 9.09, *p* < 0.01, ${\text{η}}_{\text{p}}^{\text{2}}$ = 0.23, ${\text{η}}_{\text{G}}^{\text{2}}$ = 0.014, revealed a significant difference in step size for different emotion categories, regardless of duration. Pairwise comparisons showed that, compared to neutral stimuli, step size was significantly larger for both pleasant [*t*(31) = –3.23, *p* < 0.01, Hedges g_av_ = 0.25, CL effect size = 72%] and unpleasant [*t*(31) = –3.27, *p* < 0.01, Hedges g_av_ = 0.25, CL effect size = 72%] stimuli. The data also showed a main effect for duration [*F*(2,62) = 11.27, *p* < 0.001, ${\text{η}}_{\text{p}}^{\text{2}}$ = 0.26, ${\text{η}}_{\text{G}}^{\text{2}}$ = 0.0016], showing a difference in step size for the different durations. Pairwise comparisons showed that step size was significantly larger for short durations compared to both medium [*t*(31) = 2.58, *p* < 0.05, Hedges g_av_ = 0.042, CL effect size = 68%] and long [*t*(31) = 4.34, *p* < 0.001, Hedges g_av_ = 0.10, CL effect size = 79%] durations.

### Peak Velocity

Peak velocity is plotted in Figure 3D. The ANOVA showed a main effect for duration, *F*(1.55,47.94) = 7.62, *p* < 0.01, ${\text{η}}_{\text{p}}^{\text{2}}$ = 0.20, ${\text{η}}_{\text{G}}^{\text{2}}$ = 0.0019, indicating a difference in peak velocity for the different picture durations, regardless of emotion. The pairwise comparison between the peak velocity for short and long durations was significant [*t*(31) = 3.23, *p* < 0.01, Hedges g_av_ = 0.11, CL effect size = 72%], with the longer duration resulting in a lower peak velocity in the step.

## Discussion

The aim of the present experiment was to examine the combined effects of stimulus duration and emotional content on the control of forward gait initiation. To this end, we analyzed a collection of kinematic variables that characterize key events in the COP trace with forward GI. Duration affects GI parameters of forward step initiation in multiple ways. Step size showed a clear effect; longer duration resulted in smaller steps, regardless of emotional content. In addition, we found an effect of viewing duration on the peak velocity, with shorter duration inducing higher peak velocity compared to longer duration. APA values appeared unresponsive to stimulus duration. However, there was an interesting interaction between duration and emotional content on the RTs. With increasing viewing duration RTs became longer in response to neutral images, but shorter in response to pleasant and unpleasant images. Furthermore, with short viewing durations, RT in response to neutral pictures was faster compared to both unpleasant and pleasant categories. With medium viewing time, RT in response to neutral pictures was only faster compared to unpleasant pictures. And finally, in the long viewing condition, there was no difference in RT between neutral, pleasant and unpleasant images. This showed that significant differences between pleasant and unpleasant (high-arousal) and neutral (low-arousal) stimuli were found when viewing these images for a short duration, but that these differences disappeared entirely when viewing them for a long duration.

The above effects of duration can potentially be explained by a mechanism whereby prolonged picture viewing leads to less forceful (smaller and slower) steps, which could be caused by a gradual loss of alertness with respect to the task. Reaction times in response to neutral pictures showed a slowing over time (which is consistent with the idea of loss of alertness to the task over time). However, for RT, the opposite pattern was found for both pleasant and unpleasant stimuli (i.e., significantly slower responses compared to neutral stimuli for short duration, while over longer durations the RT decreased to become faster and similar to neutral stimuli). It could be that in the short viewing duration, the emotional content (compared to the neutral content) captivated attention to such a degree that it interfered with the process of gait initiation (i.e., elevated RTs compared to neutral stimuli). When viewing time increased, however, the impact of the emotional content seemed to diminish, and thus interfered less and less with the GI process, becoming comparable to neutral stimuli.

Besides viewing duration, emotional content affected the GI parameters as well. Step size showed significant effects for different picture categories. Step size was smaller for neutral stimuli compared to both pleasant and unpleasant stimuli, regardless of viewing duration. APA amplitude showed a marginally significant trend in the data, with a larger amplitude for neutral stimuli compared to both pleasant and unpleasant stimuli, regardless of viewing duration.

An important observation is that for all four dependent variables, no significant differences were found in the direct comparison between pleasant and unpleasant stimuli. However, differences *were* found between these two emotion conditions and the neutral condition. When comparing the kinematic results to the subjective SAM ratings for both valence and arousal, it seems that arousal, but not valence, may explain this pattern found in the experiment.

With respect to valence, there were clear differences in valence ratings across the three emotion categories. Predictably, pleasant images were rated as most pleasant, unpleasant images were rated as most unpleasant, and neutral images occupied an intermediate position. However, these differences in subjective ratings were not mirrored in the GI parameters. Pleasant and unpleasant pictures yielded no statistically different effects on GI, whereas the neutral pictures differed significantly from both emotion categories.

With respect to arousal, a different picture emerged. There were again differences in arousal ratings across the three emotion categories; the neutral condition was clearly different from the two emotional conditions, in the presence of a small difference in arousal between the pleasant and unpleasant picture categories. These differences were manifest in three out of four GI parameters.

Although most studies within the field of whole-body emotional paradigms have highlighted the effects of valence on GI (e.g., Yiou et al., 2014; Stins et al., 2015), arousal seems to be a more crucial factor in explaining the current findings than valence. Returning to the results, regardless of viewing time, arousing stimuli appeared to cause larger step sizes (comparing neutral to pleasant and unpleasant stimuli) and marginally smaller APA amplitudes. Furthermore, the effect of duration on RT that was found in neutral stimuli (slower RTs over time) was completely opposite with the high-arousal emotional categories (faster RTs over time).

Hence, in contrast to most studies within this domain (e.g., Stins and Beek, 2011; Yiou et al., 2014), we found that arousal, but not valence, affects gait parameters of forward step initiation. Interestingly, similar results with regard to arousal were obtained by Naugle et al. (2011) and Horslen and Carpenter (2011).

Naugle et al. (2011) found, besides effects of valence, similar effects of arousal as observed in our data. Participants viewed neutral and both high and low arousal pleasant and unpleasant pictures. At picture offset (2–4 s after picture onset), participants walked forward on a walkway. Neutral pictures were only used to calculate percentage-wise-change scores, but the high arousal pleasant and unpleasant results were comparable to the present results. The authors found no difference between high arousal pleasant and high arousal unpleasant stimuli for any of the gait parameters, similar to the pattern in our results. The only exception was RT, which was different between the two high arousal categories, with a faster RT toward unpleasant compared to pleasant stimuli (similar to Stins et al., 2015; offset-condition).

Horslen and Carpenter (2011) performed a quiet standing task where participants were asked to observe pictures on a screen while standing on a force plate. Again, neutral pictures and both high and low arousal pleasant and unpleasant pictures were shown. The authors did not find an effect of valence on postural sway, nor an interaction between valence and arousal. However, they did find an effect of arousal: frequency of sway in the AP plane was higher in the high-arousal conditions compared to the low-arousal conditions. The authors described several physiological mechanisms that may explain the effect of arousal on postural control, e.g., a change in lower limb proprioceptive sensitivity.

Scrutinizing the literature reveals that in some cases behavioral effects may be driven by arousal instead of, or in addition to, valence. For example, Stins and Beek (2011) performed a study in which participants were instructed to step forward or backward on a force plate, depending on the valence of the picture. However, SAM ratings revealed that arousal and valence ratings were not independent. Not only were the arousal ratings of pleasant stimuli lower than those of unpleasant stimuli, valence and arousal ratings were also moderately correlated (*r* = –0.39), implying that unpleasant stimuli were also more arousing compared to pleasant stimuli. So, arousal may explain additional variance. That is, the higher RTs in response to stepping toward an unpleasant picture may in fact be caused by the arousing properties of the stimulus.

Besides highlighting the effect of arousal and duration on forward gait initiation, the present results in combination with those reported by Stins et al. (2015) brought an important methodological parameter to the fore, namely that the nature of the cue for GI may be more important than previously thought. The aim of the current experiment was to investigate the effect of viewing duration on gait initiation, under the hypothesis that evidence would be found for the MDH (faster response toward pleasant compared to unpleasant pictures) for short durations (onset condition) but that this effect would reverse for longer durations (offset condition; in line with the results of Stins et al., 2015). However, no such effect of duration was found. The general trend of the effect of viewing duration could reflect a mechanism of decrease of alertness with respect to the task, causing step size and velocity to decrease and RT in neutral stimuli to increase. Future studies should attempt to independently assess the level of attentional deployment to the stimulus, e.g., using a dual task or using unexpected auditory cues.

In addition to viewing duration, there was another difference between the onset and offset condition in the study by Stins et al. (2015). In the onset conditions, participants initiated their step at the moment the picture appeared, viewing the picture during their step initiation as well. In contrast, in the offset condition participants stepped toward a black screen after the picture had disappeared. One can imagine that participants would prefer to see a black screen compared to a mutilated face, making the black screen in fact a rewarding stimulus, potentially facilitating forward GI. If so, this would suggest that participants were engaged in a process of cognitive restructuring, whereby the black screen obtained positive properties. Future studies using the offset paradigm should take this alternate explanation into account.

On a more theoretical note, the current experiment showed that approach responses were not faster with pleasant or unpleasant stimuli for any of the viewing durations. Stins et al. (2015) only found evidence in line with the MDH in their onset condition, while their offset condition was almost identical to our long viewing duration condition. So, both viewing duration and cue appeared to modulate approach-avoidance tendencies. Note that approach and avoidance behavior has also been linked to arousal by some authors (e.g., Maki and McIlroy, 1996). These authors adopted a quiet standing paradigm, and found evidence for arousal causing forward leaning, which could be a prelude to a fight-flight response. More research is needed to test the conditions under which the MDH is applicable.

In sum, we found that both viewing duration and arousal, but not valence, influence forward step initiation parameters. There is no doubt that emotion and action are intertwined, and possibly coupled on a cognitive level, but more research is needed to uncover the mechanisms underlying these effects.

### Conflict of Interest Statement

The authors declare that the research was conducted in the absence of any commercial or financial relationships that could be construed as a potential conflict of interest.

## Footnotes

1 IAPS pictures for each picture category: *Neutral*: 2038, 2102, 2104, 2190, 2200, 2210, 2305, 2440, 7004, 7006, 7010, 7025, 7035, 7041, 7050, 7705. *Erotica*: 4607, 4608, 4609, 4611, 4625, 4643, 4645, 4649, 4651, 4653, 4659, 4660, 4670, 4680, 4687, 4694. *Sports*: 5626, 5629, 8021, 8031, 8032, 8033, 8034, 8040, 8041, 8050, 8080, 8090, 8161, 8170, 8180, 8341. *Mutilation*: 3000, 3010, 3016, 3030, 3051, 3060, 3061, 3062, 3063, 3064, 3068, 3069, 3071, 3080, 3100, 3400. *Threat*: 1120, 1201, 1300, 2683, 3500, 3530, 6022, 6200, 6210, 6230, 6250, 6313, 6350, 6510, 6550, 6560.
