# Corrigendum: Arousal and exposure duration affect forward step initiation

**DOI:** 10.3389/fpsyg.2016.00164

**Published:** 2016-02-04

**Authors:** Daniëlle Bouman, John F. Stins, Peter J. Beek

**Affiliations:** Department of Human Movement Sciences, Faculty of Behavioural and Movement Sciences, Vrije Universiteit Amsterdam, MOVE Research Institute AmsterdamAmsterdam, Netherlands

**Keywords:** emotion, motor activity, gait, reaction time, kinematics

In the original article we had added an image of a barking dog as part of Figure 1. This image was taken from the International Affective Picture System (IAPS; Lang et al., 2008). However, this was done without permission of the Center for the Study of Emotion and Attention, for which we apologize. For this reason we have now replaced Figure 1 with a modified version, whereby we removed said image.

**Figure 1:**
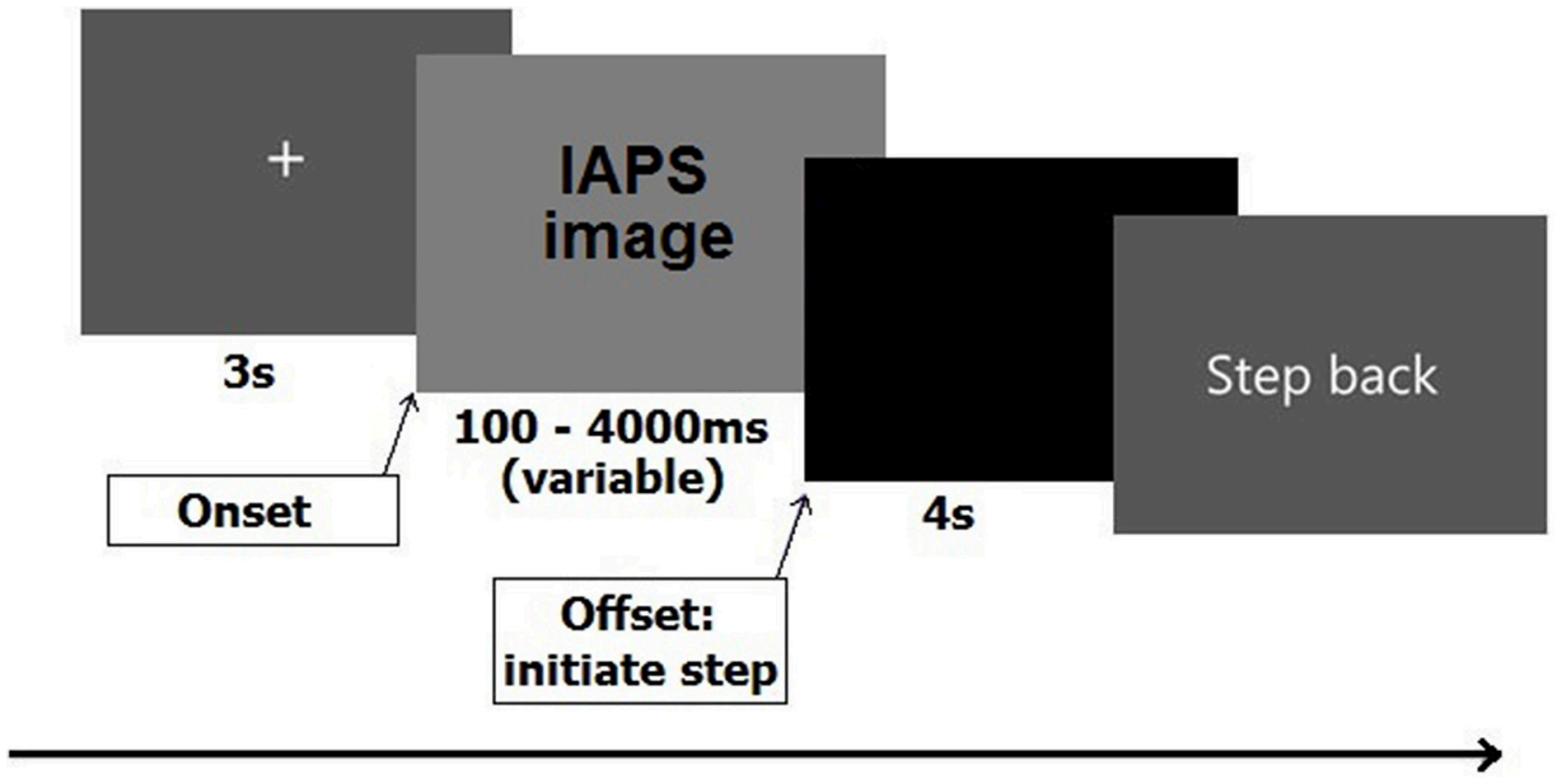
**Sequence of stimulus events for one trial**.

## Author contributions

All authors listed, have made substantial, direct and intellectual contribution to the work, and approved it for publication.

The original article was updated.

### Conflict of interest statement

The authors declare that the research was conducted in the absence of any commercial or financial relationships that could be construed as a potential conflict of interest.
